# No raw data, no science: another possible source of the reproducibility crisis

**DOI:** 10.1186/s13041-020-0552-2

**Published:** 2020-02-21

**Authors:** Tsuyoshi Miyakawa

**Affiliations:** Division of Systems Medical Science, Institute for Comprehensive Medical Science, Fujita Health University, Toyoake, Aichi 470-1192 Japan

**Keywords:** Raw data, Data fabrication, Open data, Open science, Misconduct, Reproducibility

## Abstract

A reproducibility crisis is a situation where many scientific studies cannot be reproduced. Inappropriate practices of science, such as HARKing, p-hacking, and selective reporting of positive results, have been suggested as causes of irreproducibility. In this editorial, I propose that a lack of raw data or data fabrication is another possible cause of irreproducibility.

As an Editor-in-Chief of *Molecular Brain*, I have handled 180 manuscripts since early 2017 and have made 41 editorial decisions categorized as “Revise before review,” requesting that the authors provide raw data. Surprisingly, among those 41 manuscripts, 21 were withdrawn without providing raw data, indicating that requiring raw data drove away more than half of the manuscripts. I rejected 19 out of the remaining 20 manuscripts because of insufficient raw data. Thus, more than 97% of the 41 manuscripts did not present the raw data supporting their results when requested by an editor, suggesting a possibility that the raw data did not exist from the beginning, at least in some portions of these cases.

Considering that any scientific study should be based on raw data, and that data storage space should no longer be a challenge, journals, in principle, should try to have their authors publicize raw data in a public database or journal site upon the publication of the paper to increase reproducibility of the published results and to increase public trust in science.

## Introduction

The reproducibility or replicability crisis is a serious issue in which many scientific studies are difficult to reproduce or replicate. It is reported that, in the field of cancer research, only about 20–25% [1] or 11% [2] of published studies could be validated or reproduced, and that only about 36% were reproduced in the field of psychology [3]. Inappropriate practices of science, such as HARKing (Hypothesizing After the Results are Known) [4], p-hacking [5], selective reporting of positive results and poor research design [6–8], have been proposed to be a cause of such irreproducibility. Here, I argue that a lack of raw data is another serious possible cause of irreproducibility, by showing the results of analyses on the manuscripts that I have handled over the last 2 years for *Molecular Brain*. The analysis shows that many researchers did not provide the raw data, suggesting that raw data may not exist in some cases and that the lack of data may constitute a non-negligible part of the causes of the reproducibility crisis [9]. In this editorial, I argue that making raw data openly available is not only important for reuse and data mining but also for simply confirming that the results presented in the paper are truly based on actual data. With such concept, the data sharing policy of Molecular Brain has been changed and I introduce this update.

## Raw data rarely comes out

**Fig. 1:**
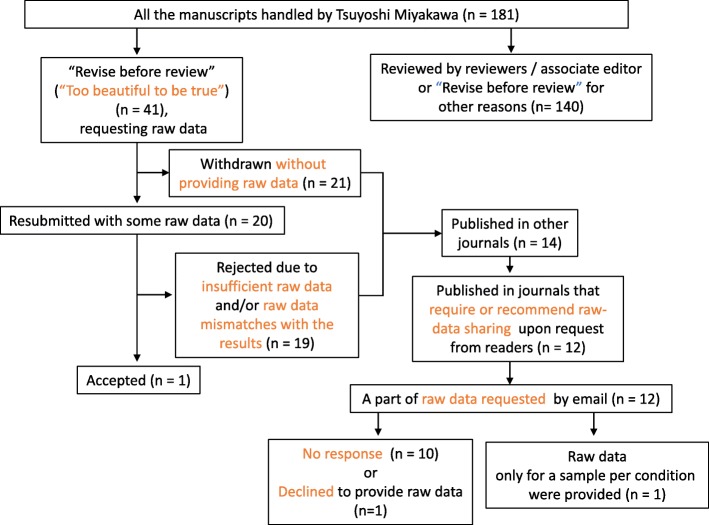
Flowchart of the manuscripts handled by Tsuyoshi Miyakawa in *Molecular Brain* from December 2017 to September 2019

As Editor-in-Chief of the journal, I have handled 180 manuscripts since early 2017 to September 2019 and have made 41 editorial decisions categorized as ‘Revise before review’, with comments asking the authors to provide raw data (Fig. 1; See Additional file 2: Table S1 for details).

Below is an example of the requests I have made to authors:

"Before proceeding, please do the following:

1. Attach raw data (all the images for entire membranes of western blotting with size markers and for staining, quantified numerical data for each sample used for statistical analyses, etc.) as supplementary materials.
2. Provide absolute *p*-values, instead of expressions like *p* < 0.05, in the results.
3. Conduct corrections for multiple tests, where necessary.

Thanks."

These comments are made when I feel that the data shown are ‘too beautiful to be true’, when error bars are too short despite a small number of samples analyzed, and/or when the effect size of experimental manipulation is huge (sometimes accompanied with complete rescue by some drug administration). Among the 41 manuscripts, 21 were withdrawn without providing raw data, indicating that simply requiring raw data drove away more than half of the manuscripts (Additional file 2: Table S1). Out of the remaining 20 cases where the authors resubmitted the manuscripts with some raw data, 19 had insufficient data. Among these, nine presented partial or no data (e.g., only one sample for a condition). In these cases, the authors were willing to provide at least some but not all of the data. In seven cases, the raw data presented by the authors did not match the data that were presented in the results. In 11 out of 18 manuscripts that included western blotting experiments, there was no indication of size markers in the images, and/or entire membrane images were not provided. In two cases, I identified evidence of image duplications and inappropriate cuts and pastes in the images provided.

Among the 41 manuscripts, only one was sent out for review and this was accepted for publication. Thus, more than 97% of the 41 manuscripts did not or could not provide appropriate raw data supporting the results shown when requested by an editor. Note that the editorial policy of *Molecular Brain* states that submission of a manuscript implies that materials described in the manuscript, including all relevant raw data, will be freely available to any scientist wishing to use them for non-commercial purposes.

Among the 40 withdrawn or rejected manuscripts, 14 were later published in other journals. Twelve journals out of those that published the 14 papers require or recommend that the authors provide raw data upon request from readers in their policies. Therefore, we sent emails and printed letters to the authors of the 12 papers in those journals requesting raw data for the results in a Figure in the papers. Ten of the authors of the 12 papers did not respond to our request. The one who responded sent us raw data only for one sample per condition, while each condition was supposed to have 6 samples. Another one who responded declined to send us raw data, for the reason that they found recently that the raw data include some other novel information. We requested just the images of western blotting membranes and I wonder how such images can contain novel information that is worth being kept confidential. The results of our requests for raw data indicate that requiring or encouraging researchers to provide raw data to readers after publication would not be quite meaningful. As of now, most journals from major publishers require or encourage the authors to do so, but do not require them to deposit raw data before publication, except for some particular types of data, according to the data availability policy of the publishers, such as Springer Nature [10], Cell Press [11] and PLOS [12]).

## Absence of raw data means the absence of science

With regard to the manuscripts that the authors withdrew independently, there are possible reasons for withdrawing after being asked to provide raw data. One possible reason is that although they actually had the raw data, the authors were not willing to gather all the raw data and upload them. It’s also possible that authors did not disclose raw data which they could use as an exclusive source for data mining to publish additional papers later. Another possible reason is that they chose journals where the disclosure of raw data is not required at the time of publication. However, the “data mining” hypothesis is unlikely for many of the authors in the cases considered here, since most of the rejected manuscripts did not contain big data that are suitable for data mining, as most of the requested data prior to peer review were images for western blotting or for tissue staining. Note that I asked not only for raw data but also for absolute *p*-values and corrections for multiple statistical tests; therefore, the possibility cannot be excluded that some of them did not wish to provide absolute p-values or to conduct corrections for multiple tests, though I do not think that these can be the primary reasons for the withdrawal. As for the ones that I rejected, it is technically possible that the insufficiency or mismatch between raw data and results are honest and careless mistakes.

In academia, these are usually the official interpretations that we make. According to COPE (Committee on Publication Ethics)‘s flowchart resource [10] on suspected fabricated data in a submitted manuscript, suspected fabrication should be investigated through contacting the author, and if necessary the relevant institution or regulatory body should be alerted so that they can initiate a full investigation. However, when reviewers or editors see such activities, we do not usually express direct concerns of possible misconduct or initiate official investigations about them, unless truly definitive evidence for misconduct exists. We have a strong tendency or custom to suppose an honest mistake, rather than to suspect a fabrication and to start an official investigation following such protocol. This is probably because the current system of scientific publication is based on the belief or the assumption that the nature of researchers is fundamentally good.

Considering the experiences that I as an Editor-in-Chief described above, a skeptic might raise the possibility that some of the raw data behind a summary graph did not exist, and that the representative image of western blot or immunostaining shown in the figure is from a limited sampling that do not accurately reflect the sample size denoted in the figure legend. At least in some of these cases described in Fig. 1, I cannot help thinking that that data did not exist from the beginning (yes, I am a skeptic). Could lackadaisical attitude towards data – spanning from data fabrication at worst to data neglect at least – have occurred in at least some cases?

We really cannot know what percentage of those manuscripts have fabricated data. Without formal investigation in all suspected cases, I can only speculate. At the same time, I was interested in how researchers on the internet would speculate with me. As such, I conducted a casual survey using Twitter in the Japanese language, asking what possible reasons researchers might have to withhold data when asked by editors before publication and when asked by readers after publication. The translation of the Twitter survey (Additional file 1: Figure S1A) is described in the Supplementary Text (Additional file 1). Approximately 53% of the 227 respondents from the life sciences field answered that they suspect more than two-thirds of the manuscripts that were withdrawn or did not provide sufficient raw data might have had fabricated the data. While this respondence from Japanese-speaking scientists is based on speculation without concrete examples, and is more about ‘gut feelings’, let us hypothesize that their estimation reflects the facts. If this is the case, out of the 40 manuscripts, the authors of 26 manuscripts or more committed data fabrication.

Then, how about 140 other manuscripts that were not considered “too beautiful to be true”? Note that I requested raw data only when I felt that the data were ‘too beautiful to be true’. More experienced and careful researchers than the authors who produce ‘too beautiful’ figures would probably make figures and results, based on non-existing data, that look more realistic so that the error bars and the effect sizes look as modest as those in real data. In such cases where the figures looked real, I did not ask the authors to provide raw data, which was not ideal but practically unavoidable under the current data availability policy of the journal that does not require but just encourages data deposition. It is likely that at least some manuscripts that were sent out for review, albeit not two-thirds as suggested by my online survey, would include some data that were not real. I conducted another twitter survey, and more than 60% of the 56 researchers in the life sciences field, who responded to the survey, thought that, among the 180 manuscripts handled by me, approximately the same number as or more than those whose data were ‘too beautiful’ due to fabrication may have made up their data in a manner that people would not pick it at face value or that the results would look realistic to experts (Additional file 1: Figure S1B). In other words, more than half of the researchers guessed that, among the ones who commit misconduct, the number of careful ones would be equal to or greater than those of careless researchers. Again, supposing that this speculation by 60% of the respondents was the case, this would mean that among the 180 manuscripts, 52 (=26 + 26) or more may involve data fabrication. A casual guess by researchers on Twitter led to a rough estimation that more than a quarter of the manuscripts submitted to our journal may include some misconduct.

A systematic review and meta-analysis of survey data estimated that 1.97% of authors admitted to have fabricated, falsified, or modified data or results at least once and, in surveys asking about the behavior of colleagues, the admission rate was 14.12% for falsification [13]. Misconduct was found to be reported more frequently by medical/pharmacological researchers than others in this systematic review [13], which is consistent with the fact that the authors of 34 out of the 40 manuscripts that did not provide raw data to Molecular Brain belonged to hospital or medical school in the analysis in this editorial. In another study in which the images from a total of 20,621 papers published in 40 scientific journals from 1995 to 2014 were visually screened, 3.8% of the published papers contained problematic figures, with at least half exhibiting features suggesting deliberate manipulation [14]. The estimation that a quarter of the manuscripts I handled may include data fabrication is greater than those previous estimates, although my estimates were just rough and casual speculation based on non-scientific and anecdotal episodes. It is unlikely that our journal, Molecular Brain, has higher incidents of such misconduct than other journals, since I, an Editor-in-Chief, have conducted relatively strict screening before review. The 14 journals that published the rejected or withdrawn manuscripts have impact factors issued from Clarivate Analytics, ranging from 2.219 to 4.658 (mean: 3.37) (Additional file 2: Table S1), and those standard journals that are well-accepted by the scientific community may have this serious problem, too. It should be noted that a positive correlation between “retraction index” and journal impact factor was reported [15], suggesting that high impact journals cannot be immune to this issue, either.

If a significant portion of submitted manuscripts already include data carelessness or fabrication, the reproducibility crisis would be due in part to the absence of raw data. It is not surprising that the results cannot be reproduced if the raw data of the studies do not exist from the beginning. In a survey that asked researchers what led to problems in reproducibility, more than 40% of the respondents chose the options, “raw data not available from original lab” or “Fraud”, as the factors that “always/often contribute” to irreproducible research [9]. This might be one of the most serious concerns in our research community in this era.

## The necessity of sharing raw data

In the current system, where we assume that every researcher is honest, and where raw data are not required to be submitted, the consequence is that fabricated data escapes scrutiny and gets published. The supposition that everyone is honest cannot be valid whilst simultaneously a situation exists in which more than half of the researchers guess that over 25% of all studies are based on non-existing data.

In a paper (cited more than 500 times) that listed recommendations for increasing replicability in psychology [16], it is noted,

As part of the submission process, journals could require authors to confirm that the raw data are available for inspection (or to stipulate why data are not available). Likewise, co-authors could be asked to confirm that they have seen the raw data and reviewed the submitted version of the paper.

Begley and Ioannidis recommend that institutions should make it a requirement that raw data be made available on request [17].

These recommendations are also based on the assumption that researchers are honest, at least to the extent that the authors will present raw data upon request. However, I imagine that, upon such a request, some of the authors might say, “Oops, hard disk got broken!” or similar. I do not think it is practical to suppose that every co-author sees and reviews all the raw data in a huge/interdisciplinary paper published in a high impact journal.

I believe that it is now time to design a system, based on such realistic reasoning of the majority of researchers, that not everyone is “honest,” replacing the “trust-me” system that is based on the traditional idealistic assumption that everyone is good.

The idea of open science/open data is needed in such a design and I propose that a custom should be commonly accepted, that sharing raw data publicly is a necessary condition for a study to be considered as scientifically sound, unless the authors have acceptable reasons not to do so (e.g., data contains confidential personal information).

In the past age of print publishing, it was technically impossible to publish all raw data due to the limitation of space. This limitation, however, has been virtually eliminated, thanks to the evolution of data storage devices and the internet.

Indeed, in 2014, the National Institutes of Health mandated researchers to share large-scale human or non-human genomic data, such as large-scale data including genome-wide association studies (GWAS), single nucleotide polymorphisms (SNP) arrays, and genome sequence, transcriptomic, epigenomic, and gene expression data (https://osp.od.nih.gov/scientific-sharing/genomic-data-sharing/). This year, the National Institute of Mental Health (NIMH) issued a data sharing policy, which requires NIMH-funded researchers to deposit all raw and analyzed data (including, but not limited to, clinical, genomic, imaging, and phenotypic data) from experiments involving human subjects into their informatics infrastructure to enable the responsible sharing and use of data collected from and about human subjects by the entire research community (https://grants.nih.gov/grants/guide/notice-files/NOT-MH-19-033.html). In 2018, it is reported that China mandated its researchers to share all scientific data in open national repositories (https://www.editage.com/insights/china-mandates-its-researchers-to-share-all-scientific-data-in-open-national-repositories/1523453996).

I believe that other countries may want to follow such a move. I propose that all journals should, in principle, try their best to have authors and institutions make their raw data open in a public database or on a journal web site upon the publication of the paper, in order to increase the reproducibility of published results and to strengthen public trust in science. Currently, the data sharing policy of Molecular Brain only “encourages” all datasets on which the conclusions of the manuscript rely to be either deposited in publicly available repositories (where available and appropriate) or presented in the main paper or additional supporting files, in machine-readable format (such as spread sheets rather than PDFs) whenever possible. Building on our existing policy, we will require, in principle, deposition of the datasets on which the conclusions of the manuscript rely from 1 March 2020. Such datasets include quantified numerical values used for statistical analyses and graphs, images of tissue staining, and uncropped images of all blot and gel results. The deposition does not have to be completed at the time of manuscript submission but the manuscripts will be accepted on the condition that such data are deposited before its publication. We could allow some exceptions, when the authors cannot make data public due to some ethical or legal reasons (eg. The data consist of confidential personal information, or proprietary data from third party). In such cases, the rational for not doing so should be clearly described in the data availability section of the manuscript and be approved by the handling and chief editors.

There are practical issues that need to be solved to share raw data. It is true that big data, such as various kinds of omics data and footage of animal behaviors, are hard to handle and to be deposited in a public database or repository and could be costly. Different researchers in different institutions may not have equal access to the use of the same level of repositories, or the skills to properly share their data. In addition, the definition of “raw data” could be an issue. For example, in mouse behavior, we are running a database to share “raw data” of mouse behaviors, but the database contains just quantified numerical text data. Ideally all the footage taken for behavior analysis should be shared, and we would like to do so when we obtain sufficient funding and infrastructure to realize such a database. The meaning of “raw data” should be discussed by the experts in each field of science and some consensus should be reached so that they can be shared in a systematic manner whereby re-analysis of the data and data mining can be conducted easily. Storage and sharing of confidential personal information on data derived from human subjects would be another challenge that needs to be overcome.

For these technical issues, institutions, funding agencies, and publishers should cooperate and try to support such a move by establishing data storage infrastructure to enable the securing and sharing of raw data, based on the understanding that “no raw data, no science.”

## Supplementary information

**Additional file 1: Figure S1.** Screen Capture of a Twitter survey conducted by Tsuyoshi Miyakawa. It should be noted that this survey was conducted in a casual manner, instead of declaring that this is a part of formal investigation. A: English translation of the question: “The following is a question on a hypothetical situation. It would be appreciated if anyone with research experience in life sciences could give an answer based on your actual experience. Among the manuscripts submitted to a journal, an editor asked the authors to submit all the raw data for 32 manuscripts in which he/she felt the data were “too beautiful.” For 15 manuscripts, only a portion of the data (e.g., only one representative data for each condition) was provided, and for another 15 manuscripts, the authors withdrew their submissions. In how many of these 30 reports do you think data fabrication occurred? Here, suppose that “raw data” means images of western blotting or immunostaining and that “too beautiful” means that the error bars are too short or the effect size is too large in view of the type of the experiments and the number of samples analyzed. Option 1: 0-10 manuscripts. Option 2: 11-20 manuscripts. Option 3: 21 or more manuscripts. Option 4: Just want to see the results or no experiences in life sciences.” B: English translation of the question: “ I would appreciate it if you could answer an additional question. Let's assume that 20 of these 30 manuscripts included data fabrication. In addition to the researchers who fabricated data that are “too beautiful to be true”, there should be some researchers who fabricated data that look realistic. How many researchers do you think are like the latter? Option 1: Less than half of the ones who fabricated data that are “too beautiful to be true” Option 2: Approximately the same number as those who fabricated data that are “too beautiful to be true” Option 3: More than double the ones who fabricated data that are “too beautiful to be true” Option 4: Just want to see the results or no experiences in life sciences. ”

**Additional file 2: Table S1. ** List of the 41 manuscripts that received editorial decision, "Revise before review”, for the reason that the results looked “too beautiful”.
