# Supplementary figures and images for: No raw data, no science: another possible source of the reproducibility crisis
Source: Mol Brain. 2020 Feb 21;13:24. doi: 10.1186/s13041-020-0552-2 (PMC7033918; doi:10.1186/s13041-020-0552-2)

Supplementary Figure 1

A


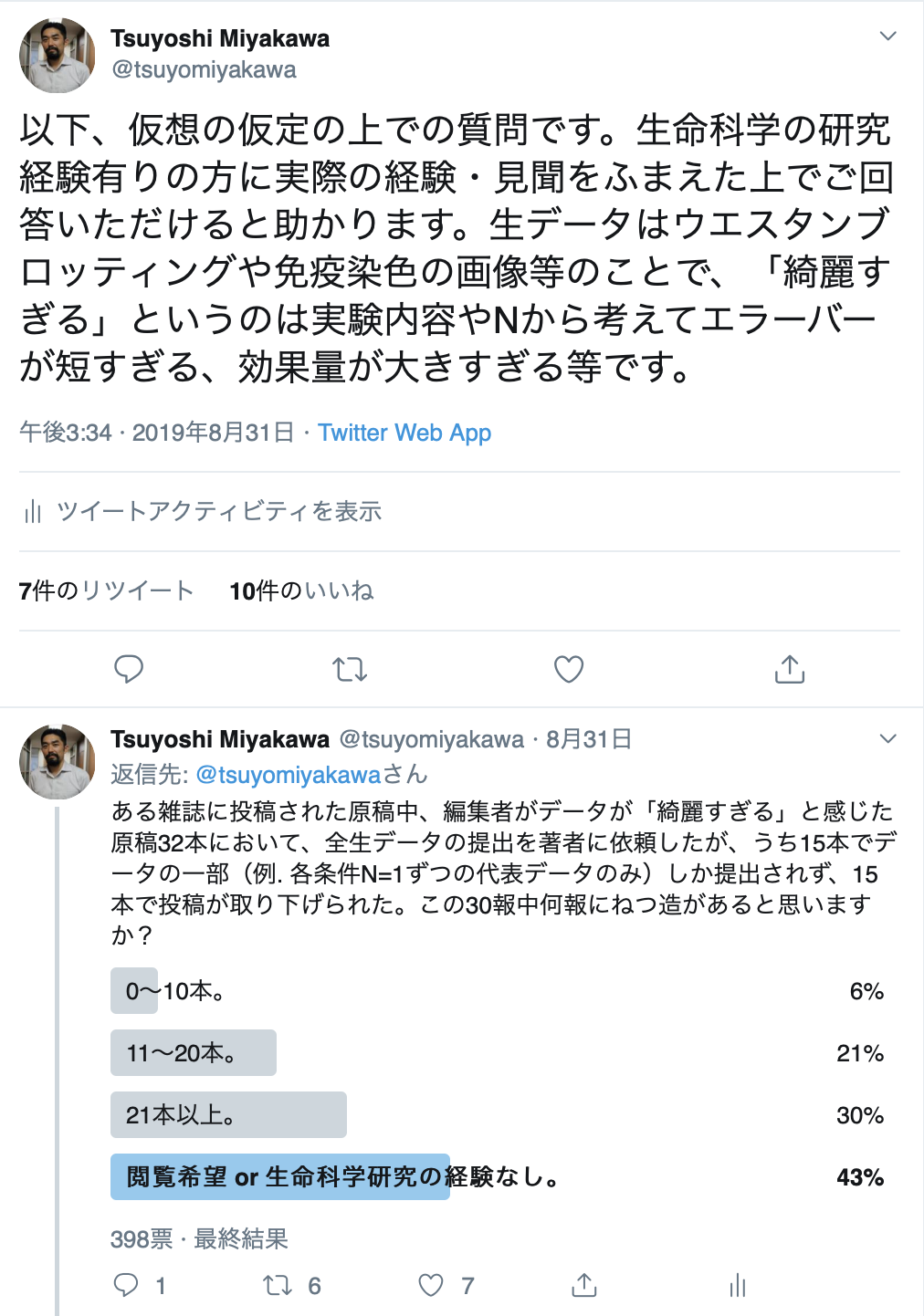


B


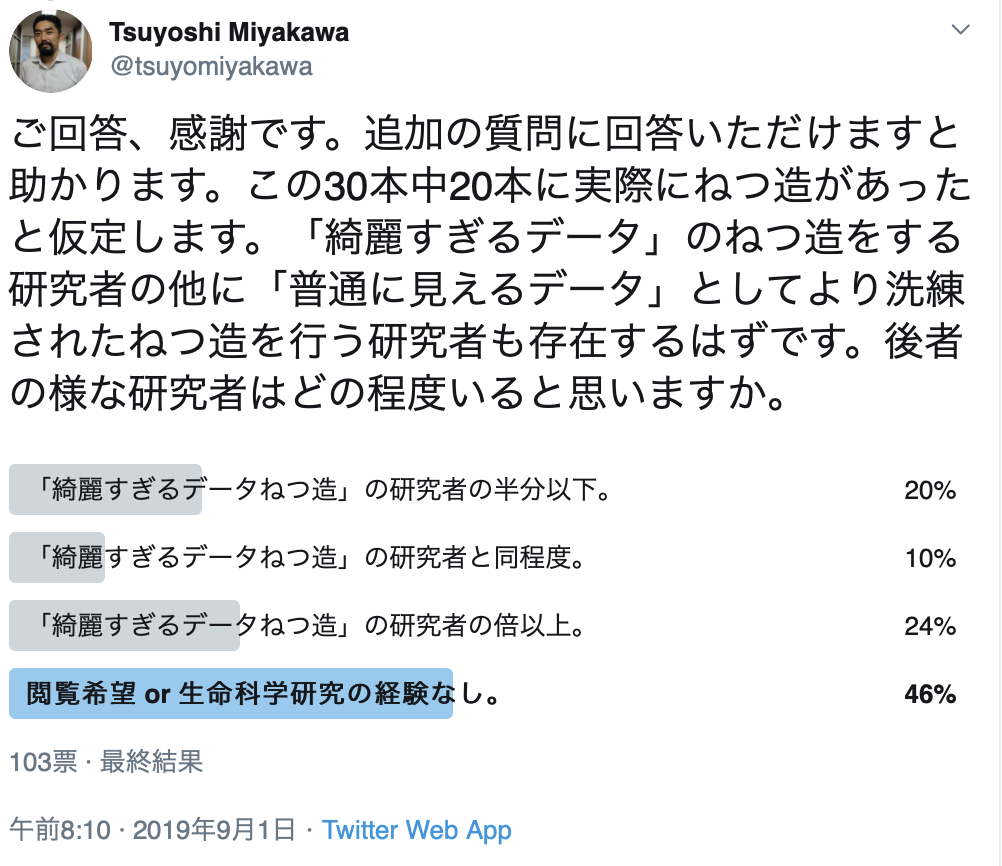

Supplement: Supplementary file 1 — Additional file 1: Figure S1. Screen Capture of a Twitter survey conducted by Tsuyoshi Miyakawa. It should be noted that this survey was conducted in a casual manner, instead of declaring that this is a part of formal investigation. A: English translation of the question: “The following is a question on a hypothetical situation. It would be appreciated if anyone with research experience in life sciences could give an answer based on your actual experience. Among the manuscripts submitted to a journal, an editor asked the authors to submit all the raw data for 32 manuscripts in which he/she felt the data were “too beautiful.” For 15 manuscripts, only a portion of the data (e.g., only one representative data for each condition) was provided, and for another 15 manuscripts, the authors withdrew their submissions. In how many of these 30 reports do you think data fabrication occurred? Here, suppose that “raw data” means images of western blotting or immunostaining and that “too beautiful” means that the error bars are too short or the effect size is too large in view of the type of the experiments and the number of samples analyzed. Option 1: 0-10 manuscripts. Option 2: 11-20 manuscripts. Option 3: 21 or more manuscripts. Option 4: Just want to see the results or no experiences in life sciences.” B: English translation of the question: “ I would appreciate it if you could answer an additional question. Let's assume that 20 of these 30 manuscripts included data fabrication. In addition to the researchers who fabricated data that are “too beautiful to be true”, there should be some researchers who fabricated data that look realistic. How many researchers do you think are like the latter? Option 1: Less than half of the ones who fabricated data that are “too beautiful to be true” Option 2: Approximately the same number as those who fabricated data that are “too beautiful to be true” Option 3: More than double the ones who fabricated data that are “too beautiful to be true” [file 13041_2020_552_MOESM1_ESM.docx]
